# An Update on Lung Function of Extremely and Very Preterm Infants in Later Life: The Role of Early Nutritional Interventions

**DOI:** 10.3390/nu15153353

**Published:** 2023-07-28

**Authors:** Despina D. Briana, Ariadne Malamitsi-Puchner

**Affiliations:** Neonatal Intensive Care Unit, 3rd Department of Pediatrics, National and Kapodistrian University of Athens Medical School, Chaidari, 12462 Athens, Greece

**Keywords:** extremely preterm neonate, very preterm neonate, bronchopulmonary dysplasia, nutrition, respiratory sequelae, chronic obstructive pulmonary disease

## Abstract

Birth occurring at ≤32 weeks’ gestation (“very preterm”) or at ≤28 weeks’ gestation (“extremely preterm”) potentially poses considerable health problems for the neonate, including respiratory sequelae, not only during the immediate newborn period, but throughout childhood and into adulthood. With the progressive improvements in neonatal care, the survival of extremely preterm and very preterm neonates has improved substantially. However, a considerable percentage of these infants suffer dysfunctions that may trigger, at some stage later in life, the onset of respiratory morbidities. The interruption of the normal development of the respiratory tract caused by preterm birth, in combination with postnatal lung injury caused by various interventions, e.g., mechanical ventilation and oxygen therapy, increases the risk ofthe development of long-term respiratory deficits in survivors. Those infants that are most affected are those who develop chronic lung disease of prematurity (also called bronchopulmonary dysplasia, BPD), but impaired lung function can develop irrespective of BPD diagnosis. Apart from indicating abnormal lung function in survivors of extreme prematurity, recent long-term follow-up studies also emphasize the crucial role of early nutritional intake as an effective strategy, which promotes lung growth and repair. This article will update the associations between extremely/very preterm birth with long-term respiratory outcomes. It will also discuss the protective effect of nutritional interventions, focusing on recently published follow-up data.

## 1. Introduction

The continuous advances in perinatal care in the last few decades (including the use of antenatal steroids and surfactant treatment), in combination with the less aggressive ventilatory support practices, have resulted in the survival of an increasing number of infants born at gestational ages (GA) ≤ 32 weeks (very preterm) or ≤28 weeks (extremely preterm). Preterm birth results in the interruption of prenatal lung development during the canalicular and saccular/early alveolar stages [1]. Early exposure to infection and inflammation, as well as mechanical ventilation and hyperoxia may cause additional damage to the immature lung [2].

A large proportion of preterm infants will eventually develop bronchopulmonary dysplasia (BPD), which is considered one of the gravest sequelae of preterm birth [3]. The “old” BPD definition provided by Northway and associates [4] >50 years ago included the characteristics of inflammation, airway smooth muscle hypertrophy, emphysema, and parenchymal fibrosis as a result of exposure to high oxygen concentration and high ventilation pressures. Since then, the cohort of premature infants, the treatment strategies of neonates, and the consequent pulmonary damage have substantially changed. The improved rates of survival among more immature infants, thanks to modern perinatal care, have resulted in the occurrence of a new BPD phenotype (disrupted lung development), which has different disease pathogenesis compared to “old” BPD (acute lung injury) [5,6]. The “new” BPD phenotype is characterized by markedly more immature lung tissue, as well as impaired alveolarization (reduced, large, thin-walled alveoli), dysmorphic pulmonary microvessel growth and less fibrosis, as compared to the “old BPD” [7]. Lung immaturity, as well as the disruption of alveolarization and the microvascular development in BPD are clinically translated into abnormal gas exchange and lung mechanics [8]. This new type of chronic lung disease of prematurity poses an additional demanding task to clinicians and researchers besides preventing lung damage, i.e., preserving normal lung tissue development. 

The exact definition of BPD has been the subject of debate and is currently influenced by the need for supplemental oxygen at 28 days of life, while the severity of the disease is determined by assessing ventilator support and the fraction of the necessary inspired oxygen at 36 weeks postmenstrual age [5,9,10]. Overall, about 30–68% of infants <28 weeks’ GA develop BPD [3,11]. This incidence is inversely related to GA and remained unchanged (or even increased) among the most premature infants due to the significant reduction in mortality and the substantial increase in the total number of infants with significant prematurity [3,11]. Large variations in BPD incidence between NICUs reflect population differences and differences in clinical treatment practices [12]. 

Previous longitudinal pulmonary function and long-term follow-up studies reported that most extremely/very preterm neonates with BPD present with a low lung function trajectory [13,14] and an increased risk for the future development of a chronic obstructive pulmonary disease (COPD)-like phenotype [15,16,17]. Furthermore, former extremely/very premature infants are predisposed to the development of other respiratory morbidities than chronic lung disease, due to factors associated with prematurity [18]. These infants present with a higher incidence of lower respiratory tract infections requiring re-hospitalizations, impaired lung mechanics, and developmental abnormalities of the airways, leading to recurrent wheezing and asthma. These sequelae may occur from early infancy throughout childhood and until adulthood [18].

Long-term respiratory outcome studies of extremely/very preterm infants are in need of constant updates due to alterations in patient population, rapid advances in the areas of pulmonary physiology and pathophysiology, as well as improvements in perinatal care practices, including nutritional interventions. In this respect, recent follow-up data highlight the crucial role of nutrition in promoting favorable long-term pulmonary outcomes in extremely/very preterm-born infants [19,20].

In the present manuscript, we review the associations between extremely/very premature birth with long-term pulmonary outcomes, with particular emphasis on the most recent data on the topic, and discuss current evidence, which emphasizes the long-term beneficial effect of nutritional interventions on lung growth and repair.

## 2. Long-Term Pulmonary Outcomes of Extremely/Very Preterm Birth 

The lung function of prematurely born infants may be compromised during childhood and adulthood; this applies, in particular, to extremely preterm infants, those experiencing BPD or those who have been exposed to mechanical ventilation during the newborn period [21]. As lung tissue grows postnatally, several parameters related to pulmonary volumes may improve. Nevertheless, pathological changes in pulmonary flows may persist throughout adolescence or into adult life [21].

### 2.1. Pulmonary Outcomes during Infancy

Several clinical/experimental studies document that preterm birth disrupts alveolarization, leading to a decrease in the gas exchange surface area of the lung and thereby causing BPD [7,8]. Furthermore, lung immaturity predisposes to respiratory morbidities other than BPD [18]. Recent data demonstrate that following preterm birth, the airway epithelium is both structurally and functionally impaired, with evidence of epithelial thickening in addition to increased inflammation and apoptosis [22]. 

Thus, primarily obstructive pulmonary abnormalities were demonstrated in extremely preterm-born infants receiving contemporary intensive care at term-equivalent age, compared with healthy full-term controls [23]. Irrespective of BPD, a strikingly abnormal pulmonary function was present at the term-equivalent age, although this was observed more intensely in the BPD group. It is, therefore, suggested that the evaluation of lung morbidity only by diagnosis of BPD or not, is probably not adequate in predicting future respiratory health [23].Similarly, in a multicenter, longitudinal birth cohort study, extremely low GA neonates receiving ambient air or on low-flow nasal cannula support had abnormal tidal breathing patterns, with no differences noted between infants with and without BPD [24]. Neither prebronchodilator nor postbronchodilator tidal breathing patterns were associated with post-discharge pulmonary disease [24]. The authors hypothesized that, besides altered respiratory mechanics, other factors (e.g., respiratory tract viral infections) [25] may be responsible for the occurrence of respiratory morbidities among preterms. 

Infants born extremely/very preterm with BPD of either degree of severity were followed-up in a longitudinal cohort study to 6 and 18 months of postnatal age. Respiratory symptoms, such as recurrent/chronic cough and wheezing, were recorded [26]. Passive lung mechanics and whole-body plethysmography, as well as tidal and raised volume rapid thoraco-abdominal compression techniques, were used to examine respiratory function. Infants with BPD presented with reduced airway function and respiratory compliance. However, mild and moderate/severe BPD differed only in terms of lower respiratory compliance in the latter, possibly as a result of delayed or altered alveolar formulation. Thus, the value of the early classification of BPD severity in predicting future lung function is considered to be very limited [26]. 

Gonçalves et al. [27] reported an increased incidence of impaired respiratory function in very preterm infants of 6–12 months of corrected age compared with same-aged full-term ones, as evaluated by forced expiratory flows using the chest compression technique, and volumes using total body plethysmography. Compromised lung function was associated with the degree of prematurity, restricted fetal growth, mechanical respiratory support and recurrent episodes of wheezing during infancy [27].

### 2.2. Pulmonary Outcomes during Childhood

Those infants who survive prematurity are at risk ofaltered pulmonary function during childhood. Only a limited number of follow-up studies have investigated the trajectories of respiratory function in extremely/very preterm-born children and showed a considerable persistent lung function compromise, which warrants follow-up and treatment consideration [28,29]. 

An earlier follow-up study investigated whether very preterm birth, BPD, and the degree of BPD severity, are predictive of future lung function in school-aged children born in the modern era, which is characterized by antenatal corticosteroids use and surfactant administration [30]. Preterm children presented with significantly decreased spirometric flow-volume parameters, as well as alveolar diffusion capacity compared with children born at term. The diagnosis of BPD was related to a marked reduction in both spirometry and diffusion capacity. Furthermore, very preterm birth and moderate/severe BPD pose an additive reduction in spirometric parameters of individuals by school age [30]. Longitudinal data on lung function in a group of extremely premature children born in the post-surfactant era also confirmed a significant airflow limitation, especially striking in BPD survivors who also demonstrated an abnormal airway growth trajectory, followed by a reduction in pulmonary function between 8 and 12 years of age [31].

Oscillatory mechanics, spirometry, multiple breath nitrogen washout, and diffusing capacity of the lung for carbon monoxide were used to test pulmonary function at 9–11 years of age in children born at term and at ≤32 weeks of gestation in the contemporary era [32]. In addition, preterm children underwent chest computed tomography (CT) and had their respiratory symptoms recorded. Compared with term controls, preterm children presented with pulmonary obstruction and hyperinflation, in addition to abnormal peripheral lung mechanics. Abnormalities in lung structure were seen in 92% of preterm children and were associated with more intense respiratory obstruction and increased incidence of severe respiratory symptoms, probably implying active lung disease [32].

A population-based cohort of children born at 22–26 weeks GA and controls born at term between 2004 and 2007 were followed-up at 6½ years of age with spirometry and impulse oscillometry [33]. It was demonstrated that a large percentage of extremely preterm-born children have impaired airway mechanics and a marked obstructive reduction in pulmonary function. A total of 40% of extremely preterm children and 15% of controls exhibited asthma-like disease. Furthermore, half of the children born at 22–24 weeks GA demonstrated a lung function below the lower limits of normal. Interestingly, severe BPD contributed to pulmonary outcomes only marginally [33].

Similarly, an earlier meta-analysis of follow-up studies including infants born preterm at 24–36 weeks GA between 1964 and 2000 demonstrated average forced expiratory volume in 1 s (FEV1) reductions of 16% in those with mild BPD and 19% in those with moderate to severe BPD [14]. The marginally significant (or even non-significant) differences in pulmonary function according to the severity of BPD in these studies suggest that respiratory deficits during childhood are probably related to the degree of prematurity and that the BPD classification is likely of limited value for the prediction of future pulmonary function as evaluated by spirometric parameters [14]. 

A recent longitudinal cohort study documented data on lung structure and function, as well as respiratory symptoms throughout childhood in a very preterm cohort born in the contemporary era [34]. It was reported that preterm children with and without BPD have declining pulmonary function trajectories from 4 to 12 years of age, with greater reductions reported in children with BPD, ongoing respiratory symptoms, and bronchial wall thickening (on chest CT) indicative of inflammation. These children may be predisposed to developing lung disease later in life [34]. Furthermore, there is evidence suggesting that lung function does not improve over time in very preterm-born children diagnosed with the severe form of either “old” or “new” BPD. By contrast, FEV1 and forced vital capacity (FVC) deteriorate from childhood to adulthood [35]. In line with these findings, a very recent study aimed to outline alterations in pulmonary function in a contemporary observational group of children born preterm whowere subsequently followedup for post-prematurity respiratory disease with pulmonary function testings [36]. Very preterm-born children demonstrated worsening obstruction in pulmonary function throughout childhood [36]. 

### 2.3. Pulmonary Outcomes in Adolescence and Young Adulthood

Pulmonary function normally increases during childhood and adolescence, reaches a peak in the mid-20s, and then gradually decreases with age [37]. This trajectory is modulated by genetic factors, antenatal events, and exposure to multiple events early in life [38]. 

A meta-analysis of cohort studies, mainly conducted during the pre-surfactant era, demonstrated that infants born either very preterm or with very low birthweight are at increased risk of not reaching their full lung growth potential during adolescence and early adulthood, a finding which suggests an increased risk of COPD in later adulthood [39]. 

Similarly, long-term data obtained in the post-surfactant era showed that survivors born either at a GA less than 28 weeks or with abirthweight less than 1000 g (particularly those who had BPD) will not achieve the normal peak of expiratory airflow by their mid-20 s [40]. The authors conclude that since nowadays many more infants who were either born at <28 weeks GA or with <1000 gbirthweight are surviving into adulthood since the 1990s, many of them will end up developing symptoms of airway obstruction later in life, especially those who experienced BPD [40]. 

A very recently published population-based study [41] reported lung function trajectories from 10 to 35 years of age in infants who were born extremely preterm. Persistent airflow obstruction was reported in early adult life and throughout the onset of the age-related decline from 25 to 35 years. Lung function after extremely preterm birth was tracked in parallel, but was significantly lower as compared to the trajectories of term-born from 10 to 35 years, including the starting age-related decline from 25 to 35 years. An existing but diminishing long-term importance of BPD was recorded, probably reflecting the recent improvements in perinatal care. However, 30% of these extremely preterm-born infants met the post-bronchodilator spirometry criteria for COPD compared with 5% of term-born infants (*p* < 0.001) [41]. 

Extremely preterm-born adolescents with “new” BPD presented with poorer lung function compared with extremely preterm-born adolescents without BPD or moderate–late preterm-born ones in a multicenter cross-sectional study [42]. Extremely preterm-born adolescents with BPD had markedly lower FEV_1_ and FVC, as well as significantly higher bronchodilator response and air-trapping. However, BPD adolescents did not demonstrate a higher incidence of asthma symptoms or a poorer quality of life, probably indicating that progress in perinatal care has favored the predominance of milder forms of chronic lung disease of prematurity [42]. In accordance, recent data demonstrated lower FEV1 in adolescents with BPD born extremely/very preterm, as compared to those without BPD, with lower FEV1 values significantly related to BPD severity [43]. The results of spirometry and impulse oscillometry measurements in the BPD compared with the non-BPD group indicate airway obstruction including involvement of peripheral airways, probably implying a predisposition to COPD in adult life in the group with severe BPD [43]. 

In line with these results, a recent prospective follow-up study reported poorer lung function in adolescents and young adults born extremely premature who experienced BPD, as compared to those without a BPD diagnosis [44]. Interestingly, 16% of subjects without BPD presented with impaired pulmonary function, suggesting that prematurity by itself has a negative impact on lung function [44]. Similar results were reported in another study which showed that spirometric parameters were worse during adulthood in those born prematurely without BPD vs. term controls [45]. 

Impaired alveolar development blocks lung-diffusing capacity. Disruption of alveolar growth due to extremely preterm birth may lead to COPD in early adulthood [46]. One controlled population-based report published in 2022 documented the longitudinal development of lung-diffusing capacity after extremely preterm birth from mid-childhood to adulthood [47]. Two cohorts born at ≤28 weeks GA or birthweight ≤ 1000 g between 1982 and 1985, as well as between 1991 and 1992 were evaluated twice, at ages 18 and 25 years and 10 and 18 years, respectively, and were compared with matched controls born at term. Extremely preterm-born individuals had impaired lung-diffusing capacity. The deficits tracked below (but in parallel) to matched full-term control groups from mid-childhood to adulthood [47]. 

Finally, a study investigating the association between prematurity and lung function with COPD in the sixth decade of life showed that severe prematurity is related to obstructive lung function deficits (including COPD) into middle age and that this effect was further aggravated bysmoking [48].

Overall, there is a paucity of longitudinal respiratory follow-up data after extremely/very preterm birth in the surfactant era, but existing evidence raises considerable concerns about the long-term pulmonary status of survivors of extremely/very preterm birth. Declines in pulmonary function are persistently observed in extremely/very preterm individuals during childhood, adolescence and adulthood and, therefore, a close targeted life-long monitoring of lung health is warranted [28,29]. However, it should be noted that premature birth is not included in authoritative statements, as a risk factor for COPD [49]. Furthermore, few pulmonologists consider early life factors in their clinical practice [49]. 

### 2.4. Differences in Pulmonary Outcomes at the Turn of the Millennium

Studies comparing respiratory outcomes in extremely preterm individuals born from 1980 to 2000 produced conflicting results, with most studies reporting improvements which parallel the remarkable recent advances in perinatal care [14,41,50,51]. 

Kotecha et al. compared findings from studies of pulmonary function conducted in the pre- and post-surfactant era, in participants aged between 5 and 23 years. One interesting finding was that the mean FEV1 for subjects with BPD had improved over time from those born in the late 1960s to those born in the early 1990s, indicating that lung impairment during the neonatal period might be less severe with ongoing improvements in neonatal care practices [14]. Another study from Norway compared respiratory health in extremely preterm-born children between 1991–1992 and 1999–2000 and showed that small airway obstruction and bronchial hyperresponsiveness were still present in children born preterm at the turn of the millennium, but outcomes were better than for children born similarly preterm in 1991–1992, especially after BPD. These data imply that better neonatal care practices improve both survival and long-term pulmonary outcomes [50].However, in a subsequent longitudinal prospective follow-up of all survivors of extremely preterm births in Victoria, Australia, during three periods (namely, 1991–1992, 1997, and 2005), no significant reduction in oxygen dependence was seen at 36 weeks and no significant improvement in lung function during childhood was detected over time, despite a marked increase in the use of less invasive ventilation after birth [51].

Finally, a very recently published population-based study addressed possible cohort effects over a period of major advances in perinatal/neonatal care [41]. Spirometry was repeated in three population-based cohorts born at ≤28 weeks GA or with birthweight ≤ 1000 g during 1982–1985, 1991–1992 and 1999–2000, and in full-term controls matched for age and gender. The deficits of these cohorts compared with term-born infants decreased with each decade of birth from 1980 to 2000 [41]. 

## 3. Nutritional Interventions and Long-Term Pulmonary Outcomes 

Prematurity is a predisposing factor for the development of lung disease. BPD is associated with high morbidity and mortality rates in survivors of severe prematurity [52]. Irrespective of BPD diagnosis, premature infants are characterized by lung immaturity at birth and deficient control of breathing [18]. They face adverse pulmonary conditions in the neonatal period and are at risk of pulmonary disorders both in the mid- as well as in the long-term, such as respiratory tract infections during infancy, recurrent wheezing and asthma during childhood and abnormalities of pulmonary function in adulthood [18]. Thus, it is imperative to identify how early exposures can be modified to decrease the risk of developing BPD or other respiratory pathology before disease progression becomes irreversible. 

Compelling evidence suggests that the alveoli continue to be formed postnatally throughout childhood and adulthood. Thus, current research should focus on further elucidation of those mechanisms responsible for postnatal lung growth, as well as the development of strategies to stimulate lung regeneration [7,53,54]. In this context, nutritional interventions have been proposed to promote postnatal alveolarization and lung growth, offering a unique opportunity to improve respiratory outcomes [19,55,56]. 

Intrauterine malnutrition is a common prenatal risk factor for BPD development when preterm birth occurs [57,58]. Malnutrition may continue postnatally since extreme prematurity poses several difficulties in providing adequate nutrient and energy intake. Both intra- and extra-uterine malnutrition may have devastating effects on the developing lung [59]. A recent study documented that very preterm infants who developed BPD received a calorie/protein ratio below that recommended for optimal growth during the first 4 weeks of life [60]. A retrospective cohort of very low birthweight infants also showed markedly lower energy and lipid intake among those who developed BPD during the first week of life [61]. Interestingly, an energy intake of less than 1778.2 kJ/kg in this time-period was related to a twofold increase in the adjusted risk of developing BPD. The authors emphasize the potential crucial role of early inclusion of lipids in parenteral nutrition, in order to promote (in combination with optimal protein content) an adequate energy intake and, therefore, to reduce the incidence of BPD [61]. Further retrospective data suggest that high fluid and low caloric intake in extremely preterm infants during the first week of life are associated with BPD severity [62]. 

In accordance, several studies pointed out extrauterine growth restriction, secondary to postnatal insufficiency in nutrient and energy intake, as a key risk factor for the development of BPD [63,64]. Due to increased energy expenditure, infants with established BPD have increased and often unmet caloric needs, compared with infants without BPD, which continue after discharge from the hospital [65]. Therefore, older studies have documented extrauterine growth restriction in BPD infants up to 12 months of age [66,67]. Interestingly, recent prospective data demonstrated poorer growth of very low birthweight infants with BPD until 36 weeks of corrected age but catch up growth accomplished by three months of corrected age, probably due to the continued improvements in nutritional practices applied to BPD infants [68]. 

Compelling data suggest a strong association between early nutrition and long-term pulmonary outcomes. In a cross-sectional study of 4- to 8-year-old children who had been born prematurely and were diagnosed with BPD, undernutrition at the age of 2 years was documented as the only factor predisposing to the development of airway distension. It was concluded that nutritional status at 2 years of age in children who were diagnosed with BPD has a significant effect on respiratory outcomes in childhood [69]. 

Up to the early 2000s, nutritional policies, applied to hospitalized neonates born with severe prematurity, resulted in significant extrauterine growth restriction [70]. In 2002, Ziegler et al. introduced a new era in the nutrition of the preterm, by reporting that “aggressive” nutrition, with increased early provision of protein and calories, resulted in better growth [71]. Nutrition of very preterm infants should ensure optimal growth as reflected in increases in body weight and head circumference. However, a recent whole-population study comprising infants born below 32 weeks of gestation in England and Wales between 2008 and 2019 showed that early postnatal weight loss has decreased, and subsequent weight gain has increased, but the weight at 36 weeks postmenstrual age was consistently below the weight of babies born at full-term. Greater weight at 36 weeks postmenstrual age was dependent on enteral nutritional intake [72]. It seems that despite significant changes in feeding policies after 2000, extrauterine growth restriction in extremely/very preterm infants remains a considerable concern. 

Current recommendations suggest an adequate nutritional strategy that includes early “aggressive” parenteral nutrition, while initiating trophic feeding and advancing to concentrated nutritive enteral feeding, i.e., providing high energy in low volume, as soon as possible [19]. Priority is given to fortified mother’s own milk, followed by fortified donor milk and preterm enriched formulas with a high density of energy and nutrients. Although evidence regarding effectiveness is limited, functional nutrient supplements, such as vitamins, zinc and iron, with a potential protective role against lung damage, are being re-evaluated. Feedings highly enriched in energy and nutrients should be given after discharge, i.e., either fortified breastmilk or enriched formula [19]. Very recently, the European Society of Pediatric Gastroenterology, Hepatology, and Nutrition (ESPGHAN) Committee of Nutrition (CoN) published an expert consensus on recommendations for the nutritional management of preterm infants with a birth weight of <1800 g. The authors emphasize the absence of strong scientific evidence in various topic areas and the need for additional high-quality research, particularly studies that evaluate long-term outcomes [73]. 

Until this day, few recent long-term studies have studied the effect of the type of early nutrition on lung function in children born preterm, but they produced interesting results [20,74,75]. In this respect, very encouraging findings regarding the impact of intensive early neonatal nutritional support up to 40–44 weeks postmenstrual age (“aggressive nutrition”) and early use of nasal continuous positive airway pressure (nCPAP) on pulmonary function of very preterm neonates at school age were recently reported [20]. This study documented no significant differences in FEV1 and FVC, as well as in the incidence of lower respiratory tract infections and associated re-hospitalization up to 8 years of age either between very preterm cases and full-term controls or between the two subgroups of preterm infants with and without BPD. It was concluded that “aggressive” nutrition and early use of early nCPAP and their beneficial effect on early postnatal growth probably contributed to normal respiratory function in the study population [20]. In a 6-year follow-up study, very preterm-born infants breastfed at hospital discharge were subsequently randomized to receive either unfortified or fortified maternal milk, whereas those infants that were not breastfed received a preterm formula until 4 months of corrected age [74]. Fractional exhaled nitric oxide, airway resistance and occlusion measurements with reversibility were performed at 6 years of age. The results of the study indicated that protein-enriched nutrition after discharge may improve lung function in very preterm-born children [74]. In a cohort study with a similar design, compared to exclusively breastfed, very preterm infants supplemented with human milk fortifier or fed exclusively a preterm formula for 4 months did not have an increased risk of developing recurrent wheezing during the 1st year of life [75]. Furthermore, a study comprising infants with BPD aged less than 36 months, demonstrated that a longer duration of breast milk intake is associated with a reduced risk of acute and chronic pulmonary morbidities, such as episodes of cough or chest congestion, a reduced need for systematic administration of steroids and fewer re-hospitalizations. The authors highlight the crucial role of prolonged breast milk consumption among preterm infants with a BPD diagnosis in terms of protection against respiratory morbidities [76]. 

Further long-term follow-up studies, with larger populations, are essential in order to elucidate the potential modification of lung function in relation to early nutrition and growth in extremely and very preterm-born children [77].Moreover, prospective research is urgently needed to investigate whether better extrauterine growth of extremely/very preterm infants achieved by application of the new ESPGHAN CoN consensus-based feeding policies [73] will positively influence their respiratory outcome, as relevant retrospective data have shown [20].

## 4. Conclusions

Although the respiratory consequences of preterm birth are well-known, they remain poorly understood. BPD remains the most frequent adverse outcome for infants born <30 weeks of GA and the most common chronic lung disease in infancy. Accumulative evidence indicates persistent abnormalities of lung function in survivors of extreme prematurity throughout childhood and into adulthood, irrespective of BPD diagnosis. Long-term follow-up studies suggest that extremely/very premature birth represents an important precursor of chronic obstructive pulmonary disease that needs to be identified by pulmonologists and targeted by researchers. Nutrient intake and nutritional practices seem to have a major impact not only on short-term respiratory morbidities, but also long-term pulmonary outcomes. Efforts should remain focused on the prevention of preterm labor, but novel research should also aim at promoting postnatal alveolarization and lung regeneration. In this context, further follow-up studies focusing on the effect of early nutrition on respiratory health and lung function outcomes of extremely/very preterm individuals are urgently needed. 

## Data Availability

Not applicable.
